# Towards Optimal Health Through Boredom Aversion Based on Experiencing Psychological Flow in a Self-Directed Exercise Regime—A Scoping Review of Recent Research

**DOI:** 10.3390/sports13060161

**Published:** 2025-05-27

**Authors:** Carol Nash

**Affiliations:** History of Medicine Program, Department of Psychiatry, Temerty Faculty of Medicine, University of Toronto, Toronto, ON M5S 1A1, Canada; carol.nash@utoronto.ca

**Keywords:** optimal health, self-direction, exercise regime, boredom, psychological flow, Csikszentmihalyi, PRIMSA-ScR, COVID-19, skill level, gender

## Abstract

Background: Optimal health requires self-direction for exercise regime consistency. Boredom may cause abandoning regular exercise. Experiencing psychological flow—a concept psychologist Csikszentmihalyi originated—may avert boredom. Method: A search of post-2020 peer-reviewed publications following the PRISMA-ScR guidelines for scoping reviews investigates the range of research on this topic. The databases searched are OVID, ProQuest, PubMed, Scopus, Web of Science, and Google Scholar. The keywords are “Csikszentmihalyi AND flow AND exercise AND boredom”. Included returns contain all the keywords. Those excluded are reviews, books, reports missing any keywords, non-English reports, reports not based on research studies, and research published before 2020. Results: Two databases returned the included results: OVID (n = 3) and Google Scholar (n = 8). Conclusions: (1) Boredom is not evident when experiencing exercise-programme psychological flow. (2) Psychological flow evolves with self-directed changes in an exercise programme. (3) Successful exercise programme modifications during COVID-19 considered the imposed limitations. (4) Exercise regimes that are neither excessive nor extreme promote optimal health. And (5) optimal health accounts for exercise skill level and gender. Additionally, cognitive bias is avertable with a research team. Studies should include the research date and location and how flow reduces boredom, permitting accurate comparisons.

## 1. Introduction

The definition of optimal health includes several factors. Some are a healthy diet [1], sufficient [2] and consistently timed sleep [3], meeting a variety of social determinants (including food stability, education, income, safety, housing, access to care, and a fair legal system) [4], lacking each of early childhood adversity, toxic stress, and racism [5], and satisfaction with body weight [6]. Most of these ingredients for optimal health are outside the control of the individual and depend on a stable and supportive social structure. They are the components recognized by the focus on eliminating health inequity of the US Advisory Committee on National Health Promotion and Disease Prevention Objectives for 2030 [7]. Yet, three of these variables are directly modifiable by the individual. These are diet, sleep, and perception of body weight. That the individual can modify them is evident, although social inequalities remain regarding each [8,9,10]. However, whether any specific individual can successfully alter these aspects is questionable [11]. The reason is that diet and body weight perception correspond [12] in their effect on optimal health and are difficult to disentangle. Moreover, combining diet, sleep, and body weight perception is necessary for optimal health [13]. In this way, the dependence of optimal health on self-direction is recognized [14].

Self-direction is the degree to which one can formulate goals and learn from previous experiences [15] in making decisions free from external control [16]. For optimal health, there is an as-yet unmentioned determinant that can be affected by self-direction to a significant extent. Physical activity is an ingredient. It can promote optimal health when the individual can explore, discover, and adapt [17]. The 1985 definition of physical activity is “any bodily movement produced by skeletal muscles that results in energy expenditure” [18]. However, the definition of physical activity recently expanded to recognize it as inherently cerebral, depending on cognitive and emotional aspects [19]. Consequently, physical activity must involve more than expending energy to produce optimal health [15]. It must also connect with the thoughts and emotions of the individual in being freely chosen, inherently social, spatially situated, and political [20]. As an experience, it must be enjoyed and connected with all significant aspects of the individual for the physical activity to promote optimal physical health. Yet, as the fourth leading risk factor for global mortality [21], physical inactivity is not equivalent to a lack of exercise. Exercise is a subset of physical activity requiring the following: (1) an ability to tolerate physical activity without physical discomfort or compromise, (2) a safe place to perform the physical activity, and (3) the motivation to engage in the physical activity [20]. As such, exercise is a self-directed physical activity [16] requiring stability in an individual’s life beyond what is necessary for physical activity [22]. The type of self-regulation necessary for self-direction is a crucial component that can be learned [23].

Perhaps the most formidable barrier to individual success in achieving optimal health through exercise is a lack of consistency in maintaining an exercise regime. One reason is that the understanding of consistency has a wide discrepancy [24]. Assessing consistency in exercise is a challenge because it represents a complex, multidimensional process [25]. Developing regular, cue-triggered routines as exercise habits is essential [26]. Consistency in this regard includes adherence to several variables: (1) a programme, (2) the length of each exercise, and (3) maintaining a designed scheme [27]. Together, they support the frequency, intensity, duration, and type of exercise [28] necessary to counteract physical inactivity [29]. Together, they are dependent on mental fitness [30]. Boredom with the programme can be the factor that halts an exercise regime, particularly for some age groups [31,32,33,34] and various forms of ill health [35,36]. A link between retaining the exercise habit and relieving boredom has been found in the individual engaging in goal adjustment [37] commensurate with their activity level [38].

A negative emotion in which the individual cannot attend to the moment or find meaning defines boredom; yet, in moderation, boredom is adaptive in promoting creativity [39]. Regarding consistent boredom, there is a link between it and depression, somatic complaints, substance abuse, obesity, and eating disorders [40]. Additionally, there was an exacerbation of psychological health sequelae of young people’s experience of boredom by the limitations of the COVID-19 pandemic [41]. Throughout the COVID-19 pandemic, studies demonstrated increases in boredom and its negative impact on mental health [42]. Therefore, it is valuable to recognize that those who exercise regularly have a lower proneness to boredom plus reduced anxiety [43]. As boredom represents an emotion, a psychological framework like flow theory is relevant regarding boredom. *Beyond Boredom and Anxiety* [44]—the seminal work by Csikszentmihalyi introducing the concept of flow—recognizes alleviating the negative aspects of boredom as an essential purpose of flow. Unlike boredom, psychological flow represents complete engagement through a positive evaluation of personal efforts regarding the challenge of a task [45].

The basis of psychological flow theory, pioneered by psychologist Mihaly Csikszentmihalyi (1934–2021 [46]) is an analysis of experiences judged as enjoyable [47] by individuals participating in various activities he described as “play-forms”. These play-forms include athletic pursuits, among others [48]. However, regarding enjoyment, the focus is on the conditions under which challenging activities are sustainable. According to Csikszentmihalyi, “flow makes us feel better in the moment, enabling us to experience the remarkable potential of the body and mind fully functioning in harmony. But what makes flow an even more significant tool is its ability to improve the quality of life in the long run” [49] (p. 63). This concentration by Csikszentmihalyi on a continuing improvement process presents why flow theory is valuable regarding the sustainability of an exercise regime and is particularly relevant for attenuating boredom [44]. Flow is a theory recognized as one of the most significant in contemporary psychology [50], directing an extensive amount of research [51] in numerous disciplines [52]. What identifies flow in an activity is that it is a self- and goal-directed process for meeting challenges guided by an individual’s interests. It depends on several elements to be optimal: (1) clear goals; (2) instantaneous feedback; (3) skills required are equal to the challenge; (4) awareness and action are integrated; (5) distractions are ignored; (6) failure is not an option; (7) no self-consciousness is involved; (8) there is a distortion of time; and (9) the engaged activity is the desired end [53].

In considering the relationships between maintaining a self-directed exercise regime, psychological flow, and boredom aversion, this study aims to conduct a scoping review following PRISMA-ScR guidelines. The intention is to investigate the range of peer-reviewed studies published since 2020 demonstrating the significance of flow for maintaining an exercise regime, particularly in alleviating the boredom that instigates abandoning it. The hypothesis is that there is a direct and inverse relationship between achieving psychological flow through self-direction and averting boredom that leads to halting an exercise regime. Since 2020, several systematic reviews have investigated psychological flow regarding exercise [54,55,56,57]. What is lacking is the relevance of flow to boredom reduction. This scoping review is significant because it is the first to investigate the range of peer-reviewed publications published during the pandemic on this topic.

## 2. Materials and Methods

### 2.1. Selection of the Review Type

The study follows the 2020 PRISMA-ScR guidelines for scoping reviews [58,59]. Pre-registration of the review protocol for this study is at osf.io/g6m7k. The internet archive link is https://osf.io/mf4d8. The registration date was 29 March 2025 (accessed on 25 May 2025). The registration DOI is https://doi.org/10.17605/OSF.IO/MF4D8. The pre-registration is at OSF Registries of the Centre for Open Science [60]. All aspects of this review are by one investigator.

The choice for this study is a scoping review rather than a systematic review and meta-analysis. A scoping review is selected in contrast to a systematic review and meta-analysis as the search and analysis method because the intent is to (1) identify the types of available evidence in a given field, (2) clarify the key concepts, (3) examine the conducting of the research, (4) identify key characteristics or factors related to the concept, and (5) identify and analyze knowledge gaps. This enumeration represents the formal definition of scoping reviews as evidence synthesis [61]. This aim contrasts with examining the study’s PICO (population, intervention, comparison, and outcome) [62]. Examining the PICO is the aim of a systematic review and meta-analysis. A research synthesis conducted by review groups with specialized skills to identify and retrieve international evidence relevant to a particular question or questions aiming to appraise and synthesize the results of a search to inform decisions regarding practice, policy, and, in some cases, further research defines a systematic review [63]. When investigating the range and depth of a topic, the advice is to conduct a scoping review rather than a systematic review and meta-analysis [62,64]. This advice guides the conclusion to conduct a scoping review.

Finding the range and depth of research on this subject published since 2020 realizes the intention of conducting a scoping review. Although flow has a longstanding history, the decision was to limit the searches to include studies from 2020 to 2024, searches conducted during October 2024. The reason is twofold. (1) Research from the previous five years is the gold standard for citations in scientific journals [65,66], and (2) the effect of the COVID-19 pandemic from 2020 to 2023 [67] on limitations to exercise regimes significantly altered them [68,69,70]. Therefore, studies published between 2020 and 2024 were considered the most relevant for achieving the purpose of this study.

The most recent comments published in 2022 regarding scoping reviews note no requirement for the number of databases to search for a scoping review [71]. Yet, there is a distinction between primary databases, which consistently return the same results, and supplementary databases, where the search results depend on the particular search [72]. The primary databases used in this search are OVID, ProQuest, PubMed, Scopus, and Web of Science. Their selection pertains to the topic searched and their high regard as databases [72]. A search in Google Scholar—a supplementary database because the results are particular to the search date [73]—extrapolated the reach of the returns. This database is recognized to outperform the coverage of either Scopus or Web of Science [73], providing the reason for its inclusion as a database to search.

### 2.2. Methods Gathering the Materials

The keywords searched common to all databases were “Csikszentmihalyi AND flow AND exercise AND boredom”. The exclusionary use of the term “Csikszentmihalyi” could exclude relevant studies exploring psychological flow but do not name the theorist. On the contrary, without this term, there was a broadening of the returns to flow in the immune system [74], flow as a process investigated by physicists [75], the PRISMA flow diagram [76], or accounts of hyperfocus that were not relevant to the theory of psychological flow originated by Csikszentmihalyi [77,78,79,80]. Since relevant to this search was only this latter understanding of psychological flow, there was no inclusion of other synonyms like “flow experience OR optimal experience”. The results are from these searches alone, not previous systematic reviews that have investigated psychological flow regarding exercise [54,55,56,57]

On 15 October 2024, the OVID search included the following Health Sciences databases: Embase Classic + Embase 1947 to 14 October 2024, APA PsycInfo 1806 to October 2024 Week 2, Ovid Healthstar 1966 to August 2024, AMED (Allied and Complementary Medicine) 1985 to September 2024, JBI EBP Database Current to October 02, 2024, Health and Psychosocial Instruments 1985 to July 2024, Journals@Ovid Full Text October 14, 2024, Ovid MEDLINE(R) ALL 1946 to 14 October 2024 (see Supplementary S1: Process for the 15 October 2024 Search of OVID, ProQuest, and PubMed, the 18 October 2024 Search of Scopus and Web of Science, and the 22 October 2024 Search of Google Scholar). All returns were from Journals@Ovid Full Text, except the first one, originating from APA Psychinfo. The search criteria were “Csikszentmihalyi AND flow AND exercise AND boredom AND English language AND 2020–2025”. There were 21 returns. The records removed before screening were one duplicate, ten literature reviews, one dissertation, and two reports that did not involve a research study. The reports excluded were two that had irrelevant information on flow, another with irrelevant information about exercise, and another with irrelevant information concerning boredom. Three represents the number of reports included for assessment.

The ProQuest search followed on 15 October 2024. The search parameters initially were “Csikszentmihalyi AND flow AND exercise AND boredom”. The addition of “English AND 2020—AND Peer reviewed AND APA PsycArticles^®^ NOT (test construction AND job performance)” resulted after noting that the first returns were primarily irrelevant. With these added parameters, the returns numbered 17. The records removed before screening included three duplicates, two literature reviews, and one report that was not a research study. There was an exclusion of nine reports lacking exercise and two that did not mention boredom. There is no inclusion of reports specific to ProQuest. All the returns considered are in Supplementary S1: Process for the 15 October 2024 Search of OVID, ProQuest, and PubMed, the 18 October 2024 Search of Scopus and Web of Science, and the 22 October 2024 Search of Google Scholar.

PubMed was the final search on 15 October 2024. The parameters searched were “Csikszentmihalyi AND flow AND exercise AND boredom”. The keywords lacked additional parameters because the initial search did not produce returns. This result is noted in Supplementary S1: Process for the 15 October 2024 Search of OVID, ProQuest, and PubMed, the 18 October 2024 Search of Scopus and Web of Science, and the 22 October 2024 Search of Google Scholar.

Scopus was the first search on 18 October 2024. The search parameters were “Csikszentmihalyi AND flow AND exercise AND boredom AND 2020–2024 AND Psychology AND Article AND English AND Flow experience AND Boredom AND Flow state AND Journal AND Final publication stage”. The initial returns were mainly irrelevant, requiring the additional parameters comparable with ProQuest. The final result was 33 returns. Yet, even with the additional keywords to narrow the search, 28 of the returns did not include exercise in the text (only in the reference list), as was the case for 5 returns regarding boredom. There was no inclusion of Scopus reports. These details are provided fully in Supplementary S1: Process for the 15 October 2024 Search of OVID, ProQuest, and PubMed, the 18 October 2024 Search of Scopus and Web of Science, and the 22 October 2024 Search of Google Scholar.

Also searched on 18 October 2024 was Web of Science. Similarly to the PubMed search three days earlier, there was no need to apply parameters additional to “Csikszentmihalyi AND flow AND exercise AND boredom” to reduce irrelevant returns as the number of returns from the initial search equaled zero. This result is noted in Supplementary S1: Process for the 15 October 2024 Search of OVID, ProQuest, and PubMed, the 18 October 2024 Search of Scopus and Web of Science, and the 22 October 2024 Search of Google Scholar.

The final search was of the Google Scholar database on 22 October 2024. Unlike the other database searches returning few results, the returns were extensive. The addition of “since 2020 AND exclude citations” to the search of “Csikszentmihalyi AND flow AND exercise AND boredom” improved accuracy. The result was 5270 returns. As a crawler-based web search engine, the most relevant reports are returned first for Google Scholar [58]. The examination was until a page of 10 results did not include a report of relevance. This process took until page 16, equaling 160 records. There was a removal of 5110 records appearing after page 16 before the screening. Also removed before screening were one record not in English, six lacking peer review, twenty literature reviews, four dissertations, and sixteen records presenting no research study. Once screening began, those unable to pass the screening process were any records without keywords in the text itself: flow (n = 4), exercise (n = 61), and boredom (n = 30). Reports not retrieved equaled one. Of the remaining reports, one included irrelevant exercise information and seven irrelevant information on boredom. The result is eight included reports. These details on the screening of the Google Scholar results are in Supplementary S1: Process for the 15 October 2024 Search of OVID, ProQuest, and PubMed, the 18 October 2024 Search of Scopus and Web of Science, and the 22 October 2024 Search of Google Scholar.

**Figure 1:**
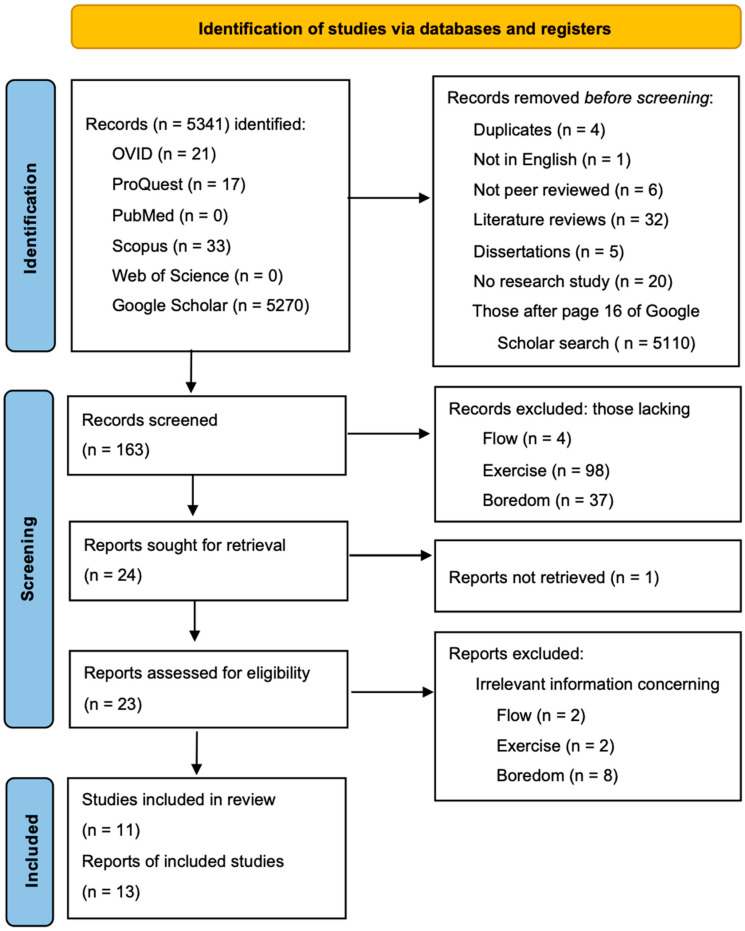
The PRISMA flow of information diagram for new systematic reviews that included searches of databases and registers only [81].

PRISMA 2020 flow diagram for new systematic reviews that included searches of databases and registers only [81] (Figure 1) represents the search of the parameter containing the keywords “Csikszentmihalyi AND flow AND exercise AND boredom” of the primary databases (with reports included): OVID (n = 3), ProQuest (n = 0), PubMed (n = 0), Scopus (n = 0), and Web of Science (n = 0), and the supplementary database, Google Scholar (n = 8), for searches conducted between 15 October 2024 and 22 October 2024, providing 11 studies. One OVID study was of 3 reports, equaling 13 reports of included studies. The PRISMA-ScR checklist outlines the entire process of this scoping review and is included as unpublished material. Following the requirements for PRISMA-ScR reporting [81], some details are not noted in the PRISMA flow diagram. Additionally, no registers were searched.

## 3. Results

The presentation of the results of the eleven studies and thirteen reports included for consideration is in three tables. These three tables follow the order and categorization of results indicated in the PRISMA-ScR Checklist [59]. This division is into (1) characteristics of sources of evidence, (2) results of individual sources of evidence, and (3) synthesis of results [59].

The first, Table 1, provides the characteristics of sources of evidence as the bibliographic details of each report. It includes the citation number, the title of the report, the authors, and the publication date. The years of publication and their number are 2024 (n = 1), 2023 (n = 2), 2022 (n = 3), and 2021 (n = 5). There is no repetition in the authors.

Table 2 provides the results of individual sources of evidence through the relevant particulars regarding the study information for each of the included returns. The studies investigating expert participants in a particular sport represent seven of the eleven, while four studied participants without expertise. Together, there are 3270 participants in these studies. The number of participants in qualitative studies is 45, with 3225 participants in quantitative studies. There were nine quantitative studies, three mixed methods, and one qualitative. Four studies reported the dates of the investigation—seven did not. There is a report of the country in every study except one. The USA represents the most studies at three, with 1887 participants. The other countries reported (and their participants) are England (n = 6, and some unspecified number of 30), Turkey (n = 532), New Zealand (some unspecified number of 30), Tunisia (n = 94), Italy (n = 1281), Germany (n = 113), and China (n = 404).

Table 3 presents the synthesis of results through the relevant content of the included reports regarding flow, exercise, and boredom for the parameters searched, identified by citation number. “Csikszentmihalyi” is not included for consideration as the name of this psychologist is a keyword searched to differentiate flow as a psychological theory rather than one concerning flow in, for example, fluid dynamics [93]. What these reports relate about the psychologist personally is irrelevant to this study.

### 3.1. Flow

Regarding psychological flow, there is agreement in the results of all studies included for assessment that flow is desirable and attainable for an exercise practice, even with limitations imposed on the regime from the 2020–2023 COVID-19 pandemic [90,92]. One study directly measured the relationship among flow, exercise, sports, and exceptional performance, finding that all were positively related [87]. How they are correlated entails several factors: (1) a challenge/skill balance, (2) action plus awareness merging, (3) the participant receiving unambiguous feedback while displaying concentration on a task, and (4) a sense of control. Notably, there are no significant gender differences [89]. This importance of challenge to an exercise programme producing flow is a relevant feature of two other studies [82,83]. One study stresses that some physical activities have a greater propensity for flow—martial arts being the most prominent example in this study [86]. What identifies a physical activity as more likely to produce flow is the commitment level of the participants to the type of exercise. Those engaged in activities they find rewarding have the most substantial inclination for flow [92]. The relationship of flow to a dedicated exercise practice is so ensconced in professional dance that those dancers participating in a study viewed flow as a vital component of their practice [85]. Similarly to the requirements of excellence in dancing, three separate reports of one study considered flow to require (1) the will to seek out information for improvement, (2) a motivation to reengage when difficulties arise, (3) persistence to reach a goal, (4) self-regulation in meeting the goal, and (5) valuing the physical activity for the exercise programme to be maintained [84]. Yet, flow is not exclusive to high-performance athletes. One study finds that flow is attainable by athletes of various levels of ability and frequency of their exercise schedules [91].

### 3.2. Exercise

The COVID-19 pandemic was recognized to significantly affect exercise programmes regarding the possibility of producing flow. Many exercises had to be performed at home, often in city apartments [90], or modified when the required exercise regime was outdoors in public [92]. One type of exercise regime that gained popularity during COVID-19 was the exergame [94], played by self-direction in following exercise instructions provided on a screen. The finding was that these exergames had longer engagement times and were more likely than traditional exercises to produce flow [82]. Several things were imperative for an exercise regime to promote flow. The first is that the participant must commit regarding their interest in the exercise [84,86]. The second for professional athletes is the flow felt during competition is sustainable in the exercise regime if it is considered immediately relevant [83]. This result included the acceptance of warm-up and team building as essential to the practice [85]. As such, maintaining these exercises by professional athletes requires adjusting the type of exercise by season and gender [89]. Flow is maintainable, and boredom is reduced [91] by self-directing sensitive adjustments into the exercise programme while accounting for participant enjoyment [88]. This result supports previous research [37,38]. However, the results of one study give reason for caution in modifying an exercise regime. When the changes are extreme or in excess, this can lead to anxiety, decreasing the wellness perception of the participant [87].

### 3.3. Boredom

It was typical to experience boredom during COVID-19 concerning maintaining an exercise regime [95]. Although helpful in this regard to produce flow, because many of the moves are repetitive, boredom was seen as a possible result of exergames when there was a lack of freedom to modify the exergames [82]. The finding was that freedom and dedication to create adaptive modifications in the structure and contents of flow-promoting activities were essential to avert boredom in a COVID-19 exercise [90]. However, during COVID-19, when poor weather, injuries, or lack of a running partner altered plans, even high-performance athletes experienced boredom [92], although the more extreme the athletic pursuit, the less likely participants were to experience boredom in their exercise routine [91]. This result may be because boredom is correlated negatively and weakly with the following types of well-being: psychological, emotional, social, and spiritual, with no physical well-being relationship [87]. Moreover, extreme athletes view themselves as achieving wellness [96]. Yet, regardless of the level of expertise, a common theme regarding boredom in considering its relationship to flow in exercise routines is that it is avertable with increasing levels of challenge [83,85,88] commensurate with the skill level of the participant [86] such that boredom is found predictable with one measure [84]. Boredom is often a catalyst for creativity to instigate change [97]. As such, it produces a better quality of experience in certain athletes than apathy or anxiety states and is considered a psychological antecedent of flow [89]—a finding supporting earlier research [39].

## 4. Discussion

This study examines the significance of flow in averting boredom by maintaining a self-directed exercise programme. It does so by considering publications between 2020 and 2024 regarding a search of “Csikszentmihalyi AND flow AND exercise AND boredom” of relevant databases. These searches returned eleven studies with thirteen reports, as one study included three reports. The only databases to produce included returns were OVID and Google Scholar. To be related are the implications, triangulation, and limitations of the results.

### 4.1. Implications

The studies included are various: they regard (1) the level of expertise in exercise, (2) the countries of investigation, (3) the number of participants, and (4) the type of studies. Given this variety, it is notable that there was no disagreement regarding the results of these reports. The importance of self-directing an exercise regime in mitigating boredom for producing optimal health is a point shared in common.

However, there are concerns regarding these included studies. There is data collection date in some, and ethics approval was before 2020 for a few. These factors might introduce time-related bias, especially as these studies are relevant to the changing limitations of the COVID-19 pandemic [98]. Moreover, the focus of most studies was either Europe or North America. Without cross-cultural comparisons, the conclusions might not be generalizable as behavioural researchers increasingly recognize that a diversity of the breadth of human experience is necessary [99,100]. However, in a 2021 report examining 1,000,000 relevant psychology publications, the incremental variance due to culture is roughly 6%, depending on all dimensions estimated simultaneously, or 1% for individual cultural dimensions across 136 bivariate relationships common to applied psychology [101]. The conclusion is that there are more similarities across cultures than differences.

An additional consideration is the quality of the assessed studies. Although all studies reported were published in peer-reviewed journals, providing sufficient details on the number of participants plus the methods employed, seven studies do not include information on when the study was conducted [83,84,85,86,87,88,89]. Although the study date is determinable for one of these studies as a result of information concerning the ethics approval date [88], five of the others include no details of the ethics approval date [84,85,86,87,89], and one of the studies assessed gained its ethics approval in 2013 [83]. As a result, the completion date of this study within the last ten years is unknown. This same study is also remiss in not stating the country of the research.

The most salient features were the following: (1) If experiencing flow, participants are not simultaneously bored [102]. However, boredom is not the opposite of flow based on the results of [89]. On the contrary, either apathy or anxiety is the opposite of flow. Instead, boredom is a precursor to change that may instigate flow. This point was recognized in 2018 [103] and 2020 [39]. (2) Boredom results from a lack of challenges [83,85,88] relevant to the skill level of the self-directed exercise participant [86]. As such, psychological flow remains in a self-directed exercise regime that involves continually increasing challenges of interest to the participant. Additionally, interest in the exercise challenge is crucial to flow maintenance [84,86]. (3) COVID-19 modifications to routine hampered the usual exercise regimes of participants. However, not only were participants able to maintain regular exercise, but they could also experience flow in doing so [90,92]. The increased use of exergames during the pandemic aided in achieving flow [82]. (4) The importance of exercise as a freely chosen, inherently social, spatially situated, and political activity connecting the thoughts and emotions of the individual was demonstrated [20]. This demonstration to produce optimal health was confirmed by the necessity of post-professional game exercise [83] and the connections developed in warm-up and team-building routines [85]. These results are in contrast to individual exercise routines that lack optimal health because they become extreme or in excess, leading to anxiety and decreasing wellness perception [87]. (5) As noted in earlier studies [104,105], the ability to engage in flow is again identified as unaffected by gender, although the types of exercises that lead to flow and diminish boredom differ [89]. That exercises and levels of expertise can differ and still lead to flow is relevant [91] and is noted elsewhere [106].

This study reviews the research findings regarding burnout avoidance in an exercise regime when experiencing psychological flow. Based on this scoping review, future studies can consider these results regarding burnout reduction theories. Theories that may be relevant to understanding the effect of psychological flow on boredom aversion include exercise motivation theory [107] involving trait mindfulness [108] or cognitive neuroscience perspectives considering control, motivation, and fatigue [109].

The more flow in athletes is understood, the more information can be precise in guiding people’s expectations of achieving flow. It is relevant that the self-regulation necessary for achieving flow can be taught [23]. That athletes have access to such guidance is pertinent, as the finding is that athletes who expect to experience flow regularly develop psychosocial difficulties when these expectations are unrealized [84,88]. There is a link between maintaining an exercise regime and relieving boredom when the individual engages in goal adjustment [37] and does so commensurate with their activity level [38]. These results provide that optimal health regarding exercise is a product of a positive achievement of self-direction regarding psychological flow rather than a negative focus on eliminating boredom as its goal. That psychological flow is possible under pandemic conditions [88,91,92,93] with exergames [83] is an encouraging finding that is likely transferable to any subsequent limitations from future pandemics.

### 4.2. Triangulation

Triangulation involves various methods to study the same phenomena [110]. The purpose is to overcome bias and validity problems in research [111], increasing confidence in the findings [112]. There are four recognized methods of triangulation: (1) method triangulation, (2) investigator triangulation, (3) theory triangulation, and (4) data source triangulation [110,111,113]. Yet, triangulation remains complicated by different terminology regarding the use of these terms [114]. This study achieved triangulation in various ways.

As a scoping review, there is only one method [115,116]. Achieving method triangulation is through the creation of (1) an examinable review protocol pre-registration, (2) a supplementary file that lists the returned results from each search, permitting any interested investigator to assess them, and (3) a preprint of an earlier version of this study [117] to which interested scholars can respond. As such, although the initial investigation is by one researcher, any researcher can re-investigate this work. This investigation aims to clarify those articles specific to the concept of psychological flow originated by Csikszentmihalyi; therefore, there has not been theory triangulation. However, psychological flow is similar to the hyperfocus described for those diagnosed with either ADHD [79,118] or autism [77]. One of these cited works indicates that hyperfocus describes an activity considered detrimental to social functioning, and psychological flow is the term when this type of focus is considered favourably [77]. Data source triangulation is achieved by searching five primary databases relevant to the topic and, to extend the research, one supplementary database.

### 4.3. Limitations

The limitations of this study regard the analysis conducted and the type of results returned.

PubMed and Web of Science returned nothing on this topic. This result is unexpected. Possibly, the reason is keyword bias. Generating and comparing keywords after reading articles is identified as an effective means to enhance the relative accuracy of keywords [119]. Using this method was not part of this search, as OVID, ProQuest, and Scopus—other primary databases—did initially produce results. Therefore, a gap in the field is not likely the reason for the lack of returns from PubMed and Web of Science. In such cases, searching the supplementary database Google Scholar is the advice [120]. Employing this method was fruitful. Nevertheless, without representation by PubMed and Web of Science as primary databases in the included reports, the sense that the review is comprehensive is weakened.

Additionally, because this is a scoping review and not a systematic review and meta-analysis [62,121], there is no evaluation of the sample sizes [122] or the validity of the measurement tools [123]. Since this work is not a systematic review and meta-analysis, there is a limitation. However, as the range of information on burnout in exercise regimes and the effect of psychological flow on averting it is the study intent, a scoping review following PRISMA-ScR guidelines is the PRISMA method advised [61,124,125,126,127].

One researcher alone completed the searches. Conducting research without a team may lead to cognitive bias [128]. Two steps counteract possible cognitive bias: (1) PRISMA-ScR procedures for scoping review [129] were followed, including completing the Preferred Reporting Items for Systematic Reviews and Meta-Analyses Extension for Scoping Reviews (PRISMA-ScR) Checklist as required for scoping reviews [59], added as non-published material. (2) A supplementary file of all searches that produced returns was created. This file is Supplementary S1. Detailed information is regarding all the returns for the primary databases. These details also concern the 160 returns initially included for assessment for Google Scholar. However, the full particulars regard only the final sixteen articles assessed before exclusions from the reports for eligibility. That detailed information on all 160 reports not included in the supplementary file is an additional limitation.

## 5. Conclusions

From this scoping review, there is confirmation of the hypothesis that there is a direct and inverse relationship between achieving psychological flow through self-direction and averting the type of boredom that leads to abandoning an exercise regime. Several conclusions follow from the results of the scoping review. These results are groupable into three categories. The first involves psychological flow, of which there are two results. (1) Boredom is not evident when experiencing exercise-programme psychological flow, and (2) psychological flow evolves with self-directed changes in an exercise programme. The next category concerns COVID-19. The result is that successful exercise programme modifications during COVID-19 considered the imposed pandemic limitations. The third category relates to the exercise regime. The two results are (1) to promote optimal health, exercise regimes should be neither excessive nor extreme, and (2) exercise skill level and gender must be accounted for to produce optimal health. These findings were particular to publications between 2020 and 2024; however, they support earlier research regarding flow, exercise, and boredom. Future research should involve working in research teams to assess the literature in a scoping review to avert cognitive bias. Additionally, the advice for future research studies is to include information on the location and date of the research and how flow reduces boredom. This advice is specifically relevant when pandemic limitations increase the boredom experienced, as they did during COVID-19.

## Figures and Tables

**Table 1:** Bibliographic details of the studies from the 15 October 2024 search of OVID as the first three presented in the order returned, with those of the 22 October search of Google Scholar following as the last eight for searches regarding “Csikszentmihalyi AND flow AND exercise AND boredom”.

#	Title	Authors (Year)
[82]	Does exergaming drive future physical activity and sport intentions?	Soltani et al., (2021)
[83]	The Aftermath of Peak Experiences: Difficult Transitions for Contact Sport Athletes	Senecal (2021)
[84]	Construction and Validation of the Interest Development Scale	Boeder et al., (2021)
[85]	A qualitative investigation of flow experience in group creativity	Łucznik et al., (2021)
[86]	Physical Activity Flow Propensity: Scale Development using Exploratory Factor Analysis with Paired Comparison Indicators	Pritikin and Schmidt (2022)
[87]	The Impact Of The Recreational Flow Experience On The Perception Of Wellness Among Individuals Engaged In Extreme Sports	Dilmaç and Tezcan (2021)
[88]	‘Living in the moment‘: mountain bikers‘ search for flow	Taylor and Carr (2023)
[89]	Relationships between flow state and motivation in junior elite tennis players: Differences by gender	Mouelhi-Guizani et al., (2023)
[90]	Finding flow in pandemic times: Leisure opportunities for optimal experience and positive mental health among Italian university students	Mangialavori et al., (2024)
[91]	24 h on the Run—Does boredom matter for ultra-endurance Athletes’ Crises?	Weich (2022)
[92]	The role of recreation specialization and self-efficacy on life satisfaction: the mediating effect of flow experience	Röglin (2021)

**Table 2:** Citation number of the eleven studies (and thirteen reports) of three returns from OVID and eight from Google Scholar for searches regarding “Csikszentmihalyi AND flow AND exercise AND boredom”, the aim of the study, research type, number of participants, and the date of the research along with its geographic location.

#	Aim	Research Type	Participants	Date—Location
[82]	Examining how the usability and playability of sport exergames affect the future intentions of participation in physical activity or actual sport	Quantitative analysis of administered questionnaire	76 healthy university students	Date not reported, ethics approval January 2013—country not reported
[83]	Examine peak experiences in sport and how such experiences in an athletic career affect the athlete’s career transition	Qualitative analysis of semi-structured interviews	Nine semistructured interviews with former male team-contact sport athletes	Date not reported nor ethics approval information—USA
[84]	Assess adult interest as a variable that can develop	Studies 1 and 2: Quantitative analysis of administered questionnaire	Three studies: 304 individuals, 484 respondents, and 103 respondents	Dates not reported nor ethics approval information—USA
[85]	Investigating the role of flow experience in a group creativity task, contemporary dance improvisation	Study 3: Mixed methods analysis of administered questionnaire and open-ended questions	Six dancers	Date not reported nor ethics approval information—England
[86]	Investigating the flow propensity of physical activities	Qualitative assessment of group creativity task	987 participants	Date not reported nor ethics approval information—USA
[87]	Determine the impact of recreational flow experience on perceived wellness among extreme sports participants	Mixed methods analysis of administered questionnaire	532 extreme sports participants	Date not reported, ethics approval 10 March 2023—Turkey
[88]	Understanding if experienced mountain bikers actively search for flow experiences	Quantitative analysis of administered questionnaire and two scales	30 mountain bikers	Date not reported nor ethics approval information—New Zealand and England
[89]	Examine relationships between differing types of motivation and the flow state and possible gender differences	Quantitative assessment of surveys	94 junior elite tennis players	Experiences provided regarding the qualifying tournament for the Arabic Championships from 26 July 2019 to 3 August 2019—Tunisia
[90]	Flow-promoting activities during COVID-19—with specific attention to leisure—were investigated	Quantitative analysis of administered questionnaire	1281 Italian university students attending courses in Health Sciences and Humanities, Social and Political Sciences	15 April 2020 and 15 May 2020—Italy
[91]	Examining the role of boredom in people who participate in ultra-endurance competitions	Mixed methods assessment of online survey and open-ended questions	113 competitors	12 June 2021–13 June 2021—Germany
[92]	Examining the relationship between recreation specialization, self-efficacy, flow experience, and life satisfaction	Quantitative analysis of the survey	404 long-distance Chinese runners	13 December to 21 December 2021—China

**Table 3:** Citation number of the eleven studies (and thirteen reports) of three returns from OVID and eight from Google Scholar for searches regarding “Csikszentmihalyi AND flow AND exercise AND boredom”, and the cumulative research findings for each of flow, exercise, and boredom.

#	Flow	Exercise	Boredom
[82]	Associated with challenge and deep concentration, not enjoyment, in exergames	Exergames promote longer engagement times than traditional forms	Many exergames consist of repetitive movements, so boredom might ensue as players begin to understand the associated game mechanics
[83]	A skills rise is proportionate to the increase in the challenge required to experience it for professionals	Promoted to ensure a continuation of flow experience following professional games	Without an increase in challenges, this is the result, proportionate to the decrease in flow
[84]	Concomitant with information seeking, motivation to reengage, persistence, self-regulation, and value	Effectiveness in promoting flow is determined by participant interest level	The Individual Interest Questionnaire predicts boredom, among other factors
[85]	Identified as a vital component of a dancer’s practice	Warm-up and team-building are essential for flow	Require adequate challenge; otherwise, experienced
[86]	Some activities offer more or less propensity for it—martial arts have the highest propensity	There must be interest and commitment to induce flow	Results when activity demands exceed skills or skills exceed demands
[87]	Found positively related to sports, exercise, and exceptional performance	When extreme or in excess can lead to anxiety, decreasing wellness perception	The boredom subdimension had no significant relationship with physical well-being but showed a negative significant relationship with the other subdimensions of well-being
[88]	Requires relatively smooth surfaces that allow and encourage the rider to achieve speed and momentum	Along with contemplation and nature experience, the most important motivations for bikers to achieve flow	Experienced most often when lacking challenge
[89]	Correlated with challenge/skill balance, action/awareness merging, unambiguous feedback, concentration on task, and sense of control with no significant gender differences	Should be modified to the tennis season and by gender	Produces a better quality of experience in tennis players than those in apathy or anxiety states as a psychological antecedent of flow
[90]	Possible during pandemic-related quarantine	Practiced within the limited spaces of city apartments	Resulted from inadaptive modifications in structure and contents of flow-promoting activities
[91]	Athletes can differ in their ability and frequency of exercise in this state	At a competitive level, reduced boredom with it, likely as a result of self-regulated regular training	Very extreme athletes report significantly lower sport-specific traits of this than other athletes
[92]	Those engaging in rewarding, specialized physical activities are more likely to experience it	There was an effect on the daily routines of runners by COVID-19	Chinese runners become this with poor weather, injuries, or lack of a running partner

## Data Availability

No new data were created.

## Supplementary Materials

The following supporting information can be downloaded at https://www.mdpi.com/article/10.3390/sports13060161/s1, Supplementary S1: Process for the 15 October 2024 Search of OVID, ProQuest, and PubMed, the 18 October 2024 Search of Scopus and Web of Science, and the 22 October 2024 Search of Google Scholar.
