# Supplementary material for: Towards Optimal Health Through Boredom Aversion Based on Experiencing Psychological Flow in a Self-Directed Exercise Regime—A Scoping Review of Recent Research
Source: Sports (Basel). 2025 May 27;13(6):161. doi: 10.3390/sports13060161 (PMC12197014; doi:10.3390/sports13060161)
Supplement: Supplementary file 1 [file sports-13-00161-s001.zip › Supplementary S1.pdf]

Supplementary S1: Process for the 15 October 2024 Search of OVID, ProQuest, and PubMed, the 18 October 2024 Search of Scopus and Web of Science, and the 22 October 2024 Search of Google Scholar

OVID—Tuesday, 15 October 2024

Search parameters:
Health Sciences
Embase Classic+Embase 1947 to 2024 October 14
APA PsycInfo 1806 to October 2024 Week 2
Ovid Healthstar 1966 to August 2024
AMED (Allied and Complementary Medicine) 1985 to September 2024
JBI EBP Database Current to October 02, 2024
Health and Psychosocial Instruments 1985 to July 2024
Journals@Ovid Full Text October 14, 2024
Ovid MEDLINE(R) ALL 1946 to October 14, 2024
Csikszentmihalyi AND flow AND exercise AND boredom
English language
2020-2025

21 returns
Excluded: 1 duplicate with Google Scholar, 10 literature reviews, 1 dissertation 2 no research study, 2 irrelevant information on flow, 1 irrelevant information on exercise, and 1 irrelevant information on boredom
3 reports included

1.
Database APA PsycInfo
Accession Number 2023-33087-135
Title
Creative work during a COVID-19 lockdown.
Year of Publication 2023
Author Clark, Taylor S.
Source Dissertation Abstracts International: Section B: The Sciences and Engineering. Vol.84(5-B),2023, pp. No Pagination Specified.
Publication Type Dissertation Abstract
Document Type Dissertation

2.
Database Journals@Ovid Full Text
Accession Number 00006211-202407000-00003.
Author Morin, Andre *; Grondin, Simon
Title
Mindfulness and time perception: A systematic integrative review.[Review]

Source Neuroscience & Biobehavioral Reviews. 162:105657, July 2024.
Document Type Review article.

3.
Database Journals@Ovid Full Text
Accession Number 00006211-202404000-00006.
Author Reich, Niklas a,b,*; Mannino, Michael c,d; Kotler, Steven c
Title
Using caffeine as a chemical means to induce flow states.[Article]
Source Neuroscience & Biobehavioral Reviews. 159:105577, April 2024.
Document Type Articles.

4.
Database Journals@Ovid Full Text
Accession Number 00000448-202404000-00007.
Author Ottiger, Beatrice 1; Veerbeek, Janne Marieke 2; Cazzoli, Dario 3; Nyffeler, Thomas 4; Vanbellingen, Tim 5
Title
The Flow State Scale for Rehabilitation Tasks: A New Flow Experience Questionnaire for Stroke Patients.[Article]
Source American Journal of Occupational Therapy. 78(2):78021800301-780218003011, March/April 2024.
Document Type Special Issue on Recovery of Function after Neurological Injury.

5.
Database Journals@Ovid Full Text
Accession Number 00011944-202338050-00004.
Author Gwyn, Wendy G. 1,; J. Cavanagh, Michael 2
Title
Adolescents' Experiences of a Developmental Coaching and Outdoor Adventure Education Program: Using Constructive-Developmental Theory to Investigate Individual Differences in Adolescent Meaning-Making and Developmental Growth.[Article]
Source Journal of Adolescent Research. 38(5):911-950, September 2023.
Document Type Articles.

6.
Database Journals@Ovid Full Text
Accession Number 01768438-202326020-00001.
Author Bursky, Mikell 1,2,; Kosuri, Mahathi 1,2,3; Walsh Carson, Kaitlin 1,2; Babad, Sara 1,2; Iskhakova, Alexandra 2; Nikulina, Valentina 1,2
Title

The Utility of Meditation and Mindfulness-Based Interventions in the Time of COVID-19: A Theoretical Proposition and Systematic Review of the Relevant Prison, Quarantine and Lockdown Literature.[Review]
Source Psychological Reports. 126(2):557-600, April 2023.
Document Type Review.

7.
Database Journals@Ovid Full Text
Accession Number 00024825-202307000-00014.
Author Jackson, S. A. a,*; Eklund, R. C. b; Gordon, A. c; Norsworthy, C. d; Mackenzie, Houge S. e; Hodge, K. f; Stephen, S. A. g
Title
Flow and outdoor adventure recreation: Using flow measures to re-examine motives for participation.[Article]
Source Psychology of Sport & Exercise. 67:102427, July 2023.
Document Type Articles.

8.
Database Journals@Ovid Full Text
Accession Number 00006832-202304000-00001.
Author Champ, Rebecca E.; Adamou, Marios; Tolchard, Barry
Title
Seeking Connection, Autonomy, and Emotional Feedback: A Self-Determination Theory of Self-Regulation in Attention-Deficit Hyperactivity Disorder.[Article]
Source Psychological Review. 130(3):569-603, April 2023.
Document Type Articles.

9.
Database Journals@Ovid Full Text
Accession Number 00006211-202303000-00020.
Author Le Roy, Barbara a,b,c,*; Martin-Krumm, Charles a,c,d; Pinol, Nathalie e; Dutheil, Frederic f,g; Trousselard, Marion a,c,h
Title
Human challenges to adaptation to extreme professional environments: A systematic review.[Article]
Source Neuroscience & Biobehavioral Reviews. 146:105054, March 2023.
Document Type Articles from the special issue on FIFTY YEARS OF SPACE NEUROSCIENCE AS PRELUDE TO EXPLORATION AND COLONIZATION edited by Jeffrey R. Alberts, Daniela Santucci. April E. Ronca.

10.
Database Journals@Ovid Full Text
Accession Number 00006832-202303000-00007.
Author Andersen, Marc Malmdorf; Kiverstein, Julian; Miller, Mark; Roepstorff, Andreas
Title
Play in Predictive Minds: A Cognitive Theory of Play.[Article]
Source Psychological Review. 130(2):462-479, March 2023.
Document Type Articles.

11.
Database Journals@Ovid Full Text
Accession Number 00004786-202210000-00001.
Author Rummel, Jan; Hagemann, Dirk; Steindorf, Lena; Schubert, Anna-Lena
Title
How Consistent Is Mind Wandering Across Situations and Tasks? A Latent State-Trait Analysis.[Article]
Source Journal of Experimental Psychology: Learning, Memory, & Cognition. 48(10):1385-1399, October 2022.
Document Type Research Articles.

12.
Database Journals@Ovid Full Text
Accession Number 00006826-202207010-00002.
Author Morris, Laurel S. 1,; Grehl, Mora M. 2; Rutter, Sarah B. 1; Mehta, Marishka 1; Westwater, Margaret L. 3
Title
On what motivates us: a detailed review of intrinsic v. extrinsic motivation.[Review]
Source Psychological Medicine. 52(10):1801-1816, July 2022.
Document Type On what motivates us: a detailed review of intrinsic v. extrinsic motivation.

13.
Database Journals@Ovid Full Text
Accession Number 00135124-202205000-00007.
Author Faigenbaum, Avery D. Ed.D., FACSM, ACSM-EP; Rebullido, Tamara Rial Ph.D., CSPS; Zaichkowsky, Leonard Ph.D.
Title
Heads-up: Effective Strategies for Promoting Mental Health Literacy in Youth Fitness Programs.[Article]
Source ACSM'S Health & Fitness Journal. 26(3):12-19, May/June 2022.
Document Type Features.

Article as PDF (8079KB)

14.
Database Journals@Ovid Full Text
Accession Number 00130470-202202000-00008.
Author Raza, Seher; Westgate, Erin C.; Buttrick, Nicholas R.; Heintzelman, Samantha J.; Furrer, Remy A.; Gilbert, Daniel T.; Libby, Lisa K.; Wilson, Timothy D.
Title
A Trade-Off Model of Intentional Thinking for Pleasure.[Article]
Source Emotion. 22(1):115-128, February 2022.
Document Type Articles.

15.
Database Journals@Ovid Full Text
Accession Number 00063119-202126120-00010.
Author Soltani, Pooya 1,2,3,4,; Figueiredo, Pedro 5,6; Vilas-Boas, Joao Paulo 1
Title
Does exergaming drive future physical activity and sport intentions?.[Article]
Source Journal of Health Psychology. 26(12):2173-2185, October 2021.
Document Type Articles.

16.
Database Journals@Ovid Full Text
Accession Number 00006211-202108000-00059.
Author Koch, Elena D. a,*; Moukhtarian, Talar R. b,c; Skirrow, Caroline d,e; Bozhilova, Natali b,g; Asherson, Philip b; Ebner-Priemer, Ulrich W. a,f
Title
Using e-diaries to investigate ADHD - State-of-the-art and the promising feature of just-in-time-adaptive interventions.[Review]
Source Neuroscience & Biobehavioral Reviews. 127:884-898, August 2021.
Document Type Review Articles.

17.
Database Journals@Ovid Full Text
Accession Number 00011905-202106000-00006.
Author Senecal, Gary
Title
The Aftermath of Peak Experiences: Difficult Transitions for Contact Sport Athletes.[Miscellaneous]
Source Humanistic Psychologist. 49(2):295-313, June 2021.
Document Type Humanistic Approaches to Sport Psychology.

18.
Database Journals@Ovid Full Text
Accession Number 01857016-202103000-00006.
Author Boeder, Jordan D.; Postlewaite, Elyse L.; Renninger, K. Ann; Hidi, Susanne E.
Title
Construction and Validation of the Interest Development Scale.[Article]
Source Motivation Science. 7(1):68-82, March 2021.
Document Type Articles.

19.
Database Journals@Ovid Full Text
Accession Number 00006211-202008000-00026.
Author Tabibnia, Golnaz
Title
An affective neuroscience model of boosting resilience in adults.[Review]
Source Neuroscience & Biobehavioral Reviews. 115:321-350, August 2020.
Document Type Review article.

20.
Database Journals@Ovid Full Text
Accession Number 01253235-202006000-00012.
Author Tierney, Laura BHlth; PhD Candidate *,1; Beattie, Elizabeth PhD, RN; Professor 1
Title
Enjoyable, engaging and individualised: A concept analysis of meaningful activity for older adults with dementia.[Article]
Source International Journal of Older People Nursing. 15(2):e12306, June 2020.
Document Type ORIGINAL ARTICLES.

21.
Database Journals@Ovid Full Text
Accession Number 00024825-202001000-00014.
Author Boudreau, Patrick a,b,*; Mackenzie, Susan Houge a,1; Hodge, Ken b
Title
Flow states in adventure recreation: A systematic review and thematic synthesis.[Article]
Source Psychology of Sport & Exercise. 46:101611, January 2020.
Document Type Articles.

ProQuest—Tuesday, 15 October 2024

Search parameters:
Csikszentmihalyi AND flow AND exercise AND boredom
English
2020–
Peer reviewed
APA PsycArticles®
NOT (test construction AND job performance)

17 returns
Excluded: 2 duplicates (with OVID), 1 duplicate with Google Scholar, 2 literature reviews, 1 no research study,
9 no exercise, 2 no boredom
0 reports included

1
Scholarly Journal
Test boredom: Exploring a neglected emotion

Goetz, Thomas; Bieleke, Maik; Yanagida, Takuya; Krannich, Maike; Roos, Anna-Lena; et al.
Journal of Educational Psychology Vol. 115, Iss. 7, (Oct 2023): 911-931.
Full Text
...Boredom...
...emotion of boredom has sparked considerable interest in research on teaching and...
...but boredom during tests and exams has not yet been examined. Based on the...

2
Scholarly Journal
A flow intervention for runners: Mixed-method evaluation

Goddard, Scott G; Stevens, Christopher J; Swann, Christian. Sport, Exercise, and Performance Psychology Vol. 13, Iss. 2, (May 2024): 144-161.
Full Text
...after in sport and exercise. This study aimed to evaluate the efficacy of a flow...
...development and evaluation of flow interventions in sport and exercise...
... 1 Flow is...

3
Scholarly Journal
How optimal is the "optimal experience"? Toward a more nuanced understanding of the relationship between flow states, attentional performance, and perceived effort

Thissen, Birte A. K; Oettingen, Gabriele. Psychology of Consciousness: Theory, Research, and Practice (Sep 12, 2024).
Full Text

...of flow. Based on Csikszentmihalyi’s (1975...
... In this study, we focus on the attentional aspect of flow,...
..., Csikszentmihalyi (1975) found perceived effortlessness...

4
Scholarly Journal
Professional status matters: Differences in flow proneness between professional and amateur contemporary musicians

Rakei, Amy; Bhattacharya, Joydeep. Psychology of Aesthetics, Creativity, and the Arts (Apr 4, 2024).
Full Text
...Csikszentmihalyi, 1997), and highly trained musicians are more prone to flow...
...Csikszentmihalyi (1990) originally introduced the concept of flow. It is...
... Professional Status Matters: Differences in Flow...

5
Scholarly Journal
Perspectives on group flow: Existing theoretical approaches and the development of the integrative group flow theory

Pels, Fabian; Kleinert, Jens. Group Dynamics: Theory, Research, and Practice Vol. 27, Iss. 4, (Dec 2023): 276-294.
Full Text
... Csikszentmihalyi (2000) , flow is typically described as consisting of nine...
...time, the flow literature (Nakamura & Csikszentmihalyi...
... Csikszentmihalyi (1975) occurs. (2) On the...

6
Scholarly Journal
Motivation to make music matters: Daily autonomous motivation, flow, and well-being in hobby musicians

Koehler, Friederike; Warth, Marco; Ditzen, Beate; Neubauer, Andreas B. Psychology of Aesthetics, Creativity, and the Arts Vol. 17, Iss. 6, (Dec 2023): 682-693.
Full Text
...(Csikszentmihalyi, 1993). The state of flow can be characterized as a mental...
...Csikszentmihalyi, 1975), many researchers have investigated the state of flow...
..., Csikszentmihalyi (1993) hypothesizes that flow...

7
Scholarly Journal
A constructive critique of the dialectical aspect of positive psychology’s second wave

Eytan, Yuval. Journal of Theoretical and Philosophical Psychology (May 23, 2024).
Full Text
...), for example, Seligman and Csikszentmihalyi (2000...
... Flow provides a natural high that, unlike artificial...
...acceptance that flow from the heart" (Ivtzan, 2016a , p...

8
Scholarly Journal
The controllosphere: The neural origin of cognitive effort

Holroyd, Clay B. Psychological Review (Feb 15, 2024).
Full Text
...), thereby facilitating the flow of information across neural modules...
...states of mental focus can be associated with so-called flow, a state of...
...(Csikszentmihályi, 1990). Such mental states are not...

9
Scholarly Journal
Everyday emotional functioning in COVID-19 lockdowns

Moeck, Ella K; Freeman-Robinson, Rachel; O'Brien, Sarah T; Woods, Jack H; Grewal, Komal K; et al. Emotion Vol. 23, Iss. 8, (Dec 2023): 2219-2230.
Full Text
...& Csikszentmihalyi, 2014). Adding to the...
...), essential work, 1 hr of exercise, or to receive or give care...
...prefer (e.g., encourage flow rather than just distraction...

10
Interventions to reduce the negative impact of online highly visual social networking site use on mental health outcomes: A scoping review
Scholarly Journal
Interventions to reduce the negative impact of online highly visual social networking site use on mental health outcomes: A scoping review

Herriman, Zoe; Taylor, Amanda M; Roberts, Rachel M. Psychology of popular media Vol. 13, Iss. 1, (Jan 2024): 111-139.
Full Text
... for PRISMA flow diagram detailing progression through the screening process...
...(Vally & D'Souza, 2019), addiction symptoms (cravings and boredom), and...
... Csikszentmihalyi...

11
Scholarly Journal

Disentangling three valence-related dimensions of emotion valuation: The good, the pleasant, and the desirable

Lee, Suhjin; McVeigh, Kieran; Garcia, Maxine; Carrillo, Vivian; Kim, Jeanie; et al. Emotion (Oct 7, 2024).
Full Text
...t tests showed that for some emotions such as happiness, calmness, and boredom,...
...& Barrett, 2001 ; Larson & Csikszentmihalyi...
...enhances enjoyment of calming (vs. exciting) amusement park rides and exercise...

12
Scholarly Journal
More direction but less freedom? How task rules affect intrinsic motivation

Mutter, Elizabeth R; Liu, Zhenxu; Gollwitzer, Peter M; Oettingen, Gabriele. Journal of Experimental Psychology: General Vol. 152, Iss. 5, (May 2023): 1484-1501.
Full Text
...Nakamura & Csikszentmihalyi, 2014). The subjective flow state manifests in...
...potential connections to flow theory (Csikszentmihalyi...
...are made to self-determination theory and flow theory. Further research is...

13
Scholarly Journal
Advancing our understanding of psychological flow: A scoping review of conceptualizations, measurements, and applications

Norsworthy, Cameron; Jackson, Ben; Dimmock, James A. Psychological Bulletin Vol. 147, Iss. 8, (Aug 2021): 806-827.
Full Text
...that definitions of flow vary immensely—even those presented by Csikszentmihalyi...
...flow researchers (including Csikszentmihalyi). Additionally, we selected 2012...
...phrase (that is, flow definition) or cited one of Csikszentmihalyi's statements...

14
Scholarly Journal
How consistent is mind wandering across situations and tasks? A latent state–trait analysis

Rummel, Jan; Hagemann, Dirk; Steindorf, Lena; Schubert, Anna-Lena. Journal of Experimental Psychology: Learning, Memory, and Cognition Vol. 48, Iss. 10, (Oct 2022): 1385-1399.
Full Text
...a technical issue or mental exercise but also one of general theoretical...
...been introduced to the literature but was inspired by the idea that a flow-like...
...Csikszentmihalyi, 2002). Additionally, we assessed WMC at both measurement...

15
Scholarly Journal
Toward a theory of conscious–nonconscious processing and getting hard (and easy) things done in everyday life

Iso-Ahola, Seppo E. Psychology of Consciousness: Theory, Research, and Practice Vol. 9, Iss. 1, (Mar 2022): 40-63.
Full Text
...exercise) are harder than others, even the seemingly easy activities can turn...
...(a) following the path of least resistance and (b) avoiding monotony, boredom,...
...70% of the U.S. population is overweight and only about 22% exercises regularly....

16
Scholarly Journal
Seeking connection, autonomy, and emotional feedback: A self-determination theory of self-regulation in attention-deficit hyperactivity disorder

Champ, Rebecca E; Adamou, Marios; Tolchard, Barry. Psychological Review Vol. 130, Iss. 3, (Apr 2023): 569-603.
Full Text
...flow in the prefrontal regions and connecting pathways to the limbic system via...
... 2007); as well as altered activity in the PFC, blood flow to limbic areas via...
...indicate that interest is associated with meaning and a sense of “flow...

17

Scholarly Journal
The aftermath of peak experiences: Difficult transitions for contact sport athletes

Senecal, Gary. The Humanistic Psychologist Vol. 49, Iss. 2, (Jun 2021): 295-313.
Full Text
...Csikszentmihalyi (1990) has provided substantial research on the study of flow...
... According to both Maslow and Csikszentmihalyi, peak...
...examination. Csikszentmihalyi does examine the possibility that flow states can...

PubMed—Tuesday, 15 October 2024

Search parameters:
Csikszentmihalyi AND flow AND exercise AND boredom

0 returns.

Scopus—Friday, 18 October 2024

Search parameters:
Csikszentmihalyi and flow and exercise and boredom
2020-2024
Psychology
Article
English
Flow experience
Boredom
Flow state
Journal
Final publication stage

33 returns
Excluded: 28 no exercise, 5 no boredom
0 reports included

	Article • *Open access*				
1	**A video-game-based method to induce states of high and low flow**<br><br>Show abstract<br><br>. Opens in a new tab.<br>Related documents	Joessel, F., Pichon, S., Bavelier, D.	Behavior Research Methods, 56(5), pp. 5128–5160	2024	1
	Article				
2	**How Job Stress Affect Flow Experience at Work: The Masking and Mediating Effect of Work-Related Rumination**<br>Show abstract<br><br>. Opens in a new tab.<br>Related documents	Feng, X.	Psychological Reports, 127(2), pp. 912–935	2024	6
	Article • *Open access*				
3	**The Effect of Mindfulness on the Promotion of Graduate Students' Scientific Research Creativity: The Chain Mediating Role of Flow**	Yao, H., Fan, Y., Duan, S.	Journal of Intelligence, 12(3), 24	2024	0

	**Experience and Creative Self-Efficacy**<br>Show abstract<br><br>. Opens in a new tab.<br>Related documents				
	Article • *Open access*				
4	**Developments and Trends in Flow Research Over 40 Years: A Bibliometric Analysis**<br>Show abstract<br><br>. Opens in a new tab.<br>Related documents	Zhang, Y., Wang, F.	Collabra: Psychology, 10(1), 92948	2024	0
	Article				
5	**Serious leisure and successful aging among elderly air volleyball players: examining the mediating role of social support and flow experience**<br>Show abstract<br><br>. Opens in a new tab.<br>Related documents	Wang, J., Tian, H.	Frontiers in Psychology, 15, 1403373	2024	0
	Article • *Open access*				
6	**Trajectories of mindfulness, flow experience, and stress during an online-based MBSR program: the moderating role of emotional exhaustion**<br>Show abstract<br><br>. Opens in a new tab.<br>Related documents	Hohnemann, C., Engel, F., Peifer, C., Diestel, S.	Frontiers in Psychology, 15, 1385372	2024	0
	Article • *Open access*				
7	**The effect of music tempo on movement flow**<br><br>Show abstract	Zhang, J., Huang, Y., Dong, Y., ... Zhu, L., Zhao, M.	Frontiers in Psychology, 15, 1292516	2024	0

. Opens in a new tab.
Related documents

	Document title	Authors	Source	Year	Citations
	Article				
8	**Investigating the Effect of Task Type and Modality on Flow Experience Among Intermediate Persian EFL Learners**<br>Show abstract<br><br>. Opens in a new tab.<br>Related documents	Ghanbaran, S., Ketabi, S., Shahnazari, M.	Journal of Psycholinguistic Research, 52(6), pp. 2835–2862	2023	0
	Article • *Open access*				
9	**Psychological Flow Training: Feasibility and Preliminary Efficacy of an Educational Intervention on Flow**<br>Show abstract<br><br>. Opens in a new tab.<br>Related documents	Norsworthy, C., Dimmock, J.A., Nicholas, J., Krause, A., Jackson, B.	International Journal of Applied Positive Psychology, 8(3), pp. 531–554	2023	3
	Article • *Open access*				
10	**A Playful Way to Promote Team Flow: Evaluation of a Positive Psychological Board Game for Team Building**<br>Show abstract<br><br>. Opens in a new tab.<br>Related documents	Kloep, L., Helten, A.-L., Peifer, C.	International Journal of Applied Positive Psychology, 8(2), pp. 405–427	2023	1
	Article • *Open access*				
11	**Authentic leadership and employee resilience during the COVID-19: The role of flow, organizational identification, and trust**<br>Show abstract<br><br>. Opens in a new tab.<br>Related documents	Mao, Y., Kang, X., Lai, Y., ... Ma, J., Bonaiuto, F.	Current Psychology, 42(23), pp. 20321–20336	2023	9

12	Article • *Open access*<br>**Is all mental effort equal? The role of cognitive demand-type on effort avoidance**<br>Show abstract<br>. Opens in a new tab.<br>Related documents	Embrey, J.R., Donkin, C., Newell, B.R.	Cognition, 236, 105440	2023	2
13	Article<br>**Influence of competition-outcome feedback in video games on players' flow experience**<br>Show abstract<br>. Opens in a new tab.<br>Related documents	Zhang, Y., Liang, Q., Wang, F.	Current Psychology, 42(21), pp. 17583–17594	2023	2
14	Article<br>**Exploring the Effect of Different Hints on Flow State in Virtual Reality**<br>Show abstract<br>. Opens in a new tab.<br>Related documents	Palombini, E.	Annual Review of CyberTherapy and Telemedicine, 21, pp. 63–68	2023	0
15	Article • *Open access*<br>**Test Boredom: Exploring a Neglected Emotion**<br>Show abstract<br>. Opens in a new tab.<br>Related documents	Goetz, T., Bieleke, M., Yanagida, T., ... Lipnevich, A.A., Pekrun, R.	Journal of Educational Psychology, 115(7), pp. 911–931	2023	5
16	Article • *Open access*<br>**Predictors of flow state in performing musicians: an analysis with the logistic regression method**	Moral-Bofill, L., López de la	Frontiers in Psychology, 14, 1271829	2023	1

	Show abstract<br><br>. Opens in a new tab.<br>Related documents	Llave, A., Pérez-Llantada, M.C.			
17	Article • *Open access*<br>**The Effects of Emotional Labor on Work Strain and Nonwork Strain Among Dancers: A Person-Centered Approach**<br>Show abstract<br><br>. Opens in a new tab.<br>Related documents	Liu, X., He, T., Yu, S., Duan, J., Gao, R.	Psychology Research and Behavior Management, 16, pp. 3675–3685	2023	2
18	Article • *Open access*<br>**The Relationship Between Trait Mindfulness and Well-Being in College Students: The Serial Mediation Role of Flow Experience and Sports Participation**<br>Show abstract<br><br>. Opens in a new tab.<br>Related documents	Lin, P.	Psychology Research and Behavior Management, 16, pp. 2071–2083	2023	6
19	Article • *Open access*<br>**Distal and proximal motivational processes related to flow experience: Investigating the role of implicit motives, affective and cognitive preferences, and perceived abilities**<br>Show abstract<br><br>. Opens in a new tab.<br>Related documents	Schiepe-Tiska, A., Schattke, K., Seeliger, J., Kehr, H.M.	Current Psychology, 42(2), pp. 1002–1012	2023	3
20	Article • *Open access*<br>**Consumed by Boredom: Food Choice Motivation and Weight**	Van Tilburg, W.A.P., Pekrun, R., Igou, E.R.	Behavioral Sciences, 12(10), 366	2022	4

	**Changes during the COVID-19 Pandemic**<br>Show abstract<br><br>. Opens in a new tab.<br>Related documents				
	Article • *Open access*				
21	**Development of Flow State Self-Regulation Skills and Coping With Musical Performance Anxiety: Design and Evaluation of an Electronically Implemented Psychological Program**<br>Show abstract<br><br>. Opens in a new tab.<br>Related documents	Moral-Bofill, L., López de la Llave, A., Pérez-Llantada, M.C., Holgado-Tello, F.P.	Frontiers in Psychology, 13, 899621	2022	7
	Article				
22	**Optimal psychological states in advanced climbers: Antecedents, characteristics, and consequences of flow and clutch states**<br>Show abstract<br><br>. Opens in a new tab.<br>Related documents	Boudreau, P., Houge Mackenzie, S., Hodge, K.	Psychology of Sport and Exercise, 60, 102155	2022	11
	Article • *Open access*				
23	**Achieving Flow: An Exploratory Investigation of Elite College Athletes and Musicians**<br>Show abstract<br><br>. Opens in a new tab.<br>Related documents	Antonini Philippe, R., Singer, S.M., Jaeger, J.E.E., Biasutti, M., Sinnett, S.	Frontiers in Psychology, 13, 831508	2022	8
	Article • *Open access*				
24	**Mindfulness meditation experiences of novice practitioners**	Osin, E.N., Turilina, I.I.	Applied Psychology: Health and Well-	2022	10

	**in an online intervention: Trajectories, predictors, and challenges**<br>Show abstract<br>. Opens in a new tab.<br>Related documents		Being, 14(1), pp. 101–121		
	Article • *Open access*				
25	**Effects of Professional Virtual Community Attributes and Flow Experience on Information Sharing Among Chinese Young Netizens: A Fuzzy-Set QCA**<br>Show abstract<br>. Opens in a new tab.<br>Related documents	Zhi, H., Zhang, D.	Psychology Research and Behavior Management, 15, pp. 3497–3511	2022	0
	Article • *Open access*				
26	**The Temporal Dynamics of Opportunity Costs: A Normative Account of Cognitive Fatigue and Boredom**<br>Show abstract<br>. Opens in a new tab.<br>Related documents	Agrawal, M., Mattar, M.G., Cohen, J.D., Daw, N.D.	Psychological Review, 129(3), pp. 564–585	2022	31
	Article • *Open access*				
27	**Stop and Go, Where is My Flow? How and When Daily Aversive Morning Commutes are Negatively Related to Employees' Motivational States and Behavior at Work**<br>Show abstract<br>. Opens in a new tab.<br>Related documents	Gerpott, F.H., Rivkin, W., Unger, D.	Journal of Applied Psychology, 107(2), pp. 169–192	2022	40
	Article • *Open access*				
28	**Flow as a Key Predictor of Subjective Well-Being Among**	Wu, J., Xie, M., Lai, Y., Mao, Y., Harmat, L.	Frontiers in Psychology, 12, 743906	2021	18

Chinese University Students: A Chain Mediating Model
Show abstract

. Opens in a new tab.
Related documents

	Document title	Authors	Source	Year	Cited by
	Article • *Open access*				
29	**How achievement motive enactment shapes daily flow experience and work engagement: The interplay of personality systems** Show abstract . Opens in a new tab. Related documents	Digutsch, J., Diestel, S.	Motivation and Emotion, 45(5), pp. 557–573	2021	4
	Article • *Open access*				
30	**The Effect of Modeling on Self-Efficacy and Flow State of Adolescent Athletes Through Role Models** Show abstract . Opens in a new tab. Related documents	Lee, S., Kwon, S., Ahn, J.	Frontiers in Psychology, 12, 661557	2021	10
	Article • *Open access*				
31	**Task Enjoyment as an Individual Difference Construct** Show abstract . Opens in a new tab. Related documents	Czikmantori, T., Hennecke, M., Brandstätter, V.	Journal of Personality Assessment, 103(6), pp. 818–832	2021	3
	Article				
32	**The preconditions and event-related potentials correlates of flow experience in an educational context** Show abstract	Wang, S., Wang, T., Chen, N., Luo, J.	Learning and Motivation, 72, 101678	2020	5

. Opens in a new tab.
Related documents

	Article				
33	**Beyond challenge-seeking and skill-building: Toward the lifespan developmental perspective on flow theory**	Tse, D.C.K., Nakamura, J., Csikszentmihalyi, M.	Journal of Positive Psychology, 15(2), pp. 171–182	2020	28

Web of Science—Tuesday, 18 October 2024

Search parameters:
Csikszentmihalyi AND flow AND exercise AND boredom

0 returns.

Google Scholar Tuesday, 22 October 2023

Search parameters:
Csikszentmihalyi, flow, exercise, boredom
since 2020
exclude citations

5270 returns
Searched until a page of 10 returns lacked any of flow, exercise, or boredom—16 pages searched, equaling 160 reports—5110 were removed before screening
Records removed before screening are the following
Not in English 1
Not peer reviewed 6
Literature reviews 20
Dissertations 4
No research study 17

No flow 4
No exercise 61
No boredom 30

Not retrieved 1

This list represents the 16 remaining articles after the removal of those excluded by examining returned information. The following are excluded by examining the full articles.

Excluded: irrelevant information on 1 exercise, 7 boredom
8 reports included

1.
[Exploring the relationship between smartphone activities, **flow** experience, and **boredom** in free time]
L Leung - Computers in Human Behavior, 2020 - Elsevier
… Pioneered by **Csikszentmihalyi** in the 1970s, **flow** experience … activities (**Csikszentmihalyi**, 1990, **Csikszentmihalyi**, 2000). … rarely participate in sports and **exercise** and often lack physical …

2.
[A qualitative investigation of **flow** experience in group creativity]
K Łucznik, J May, E Redding - Research in Dance Education, 2021 - Taylor & Francis
… findings by (Moneta and **Csikszentmihalyi** Citation1996, 227) that associated **flow** with 'a … adequate challenge, otherwise they will experience **boredom**, when challenges are too low, …

3.
[Physical Activity **Flow** Propensity: Scale Development using Exploratory Factor Analysis with Paired Comparison Indicators]
JN Pritikin, KM Schmidt - International Journal of Applied Positive …, 2022 - Springer
… studied **flow** wherever it happened (eg, **Csikszentmihalyi** & … demands then anxiety or **boredom** may result, respectively. … We **exercised** considerable discretion in merging and splitting …

4.
[How optimal is the "optimal experience"? Toward a more nuanced understanding of the relationship between **flow** states, attentional performance, and perceived effort …]
BAK Thissen, G Oettingen - Psychology of Consciousness: Theory …, 2024 - psycnet.apa.org
… In this study, we focus on the attentional aspect of **flow**, since **Csikszentmihalyi** emphasized it as the core of the experience, describingflow as "a state of concentration so focused that it …

5.
[The Impact Of The Recreational **Flow** Experience On The Perception Of Wellness Among Individuals Engaged In Extreme Sports]
E Dilmaç, N Tezcan - Journal of Basic and Clinical Health Sciences, 2021 - dergipark.org.tr
… **Csikszentmihalyi** defines the **flow** experience as a holistic state … the **boredom** channel to the
acquaintance or **flow** channels. It … A review of scientific progress in **flow** in sport and **exercise**: …

6.
[The **Flow** State Scale for Rehabilitation Tasks: A new **flow** experience questionnaire for stroke patients]

B Ottiger, JM Veerbeek, D Cazzoli… - … American Journal of …, 2024 - research.aota.org
… **flow** questionnaire that can be validly applied to stroke patients when they perform **exercises** … that could elicit three psychological states: **flow**, anxiety, or **boredom**. In this population, the …

7.

The effect of leisure involvement on place attachment: **Flow** experience as mediating role

H Tao, Q Zhou, D Tian, L Zhu - Land, 2022 - mdpi.com
… that were very purposeful, **Csikszentmihalyi** [23] found that they kept engaging in these activities without getting **bored** because they had an optimal experience, known as "**flow**", which …

8.

[PDF] THE **FLOW** EXPERIENCE IN LEISURE AMONG UNDERGRADUATS AND ITS INFLUENCING FACTORS

Y Hou, Y Jiang - iigdpublishers.com 2024
… and physical energy and the **exercise** of physiological and … "**flow** experience" was first proposed by **Csikszentmihalyi**, … **bored**, and hinder the production of leisure fluency experience. …

9.

'Living in the moment': mountain bikers' search for **flow**

S Taylor, A Carr - Annals of Leisure Research, 2023 - Taylor & Francis
… **Csikszentmihalyi** was one of the first researchers to explore the human drive for experiences beyond the everyday. His 1975 book Beyond **Boredom** and Anxiety alluded to some of the …

10.

Relationships between **flow** state and motivation in junior elite tennis players: Differences by gender

S Mouelhi-Guizani, S Guinoubi… - … Journal of Sports …, 2023 - journals.sagepub.com
… antecedents of **flow** in competitive tennis players found that players
in **flow** or **boredom** states
… nine theorized dimensions of **flow**, as described by **Csikszentmihalyi** 21 and supported in …

11.

Finding **flow** in pandemic times: Leisure opportunities for optimal experience and positive mental health among Italian university students

S Mangialavori, M Bassi… - Journal of Leisure …, 2024 - Taylor & Francis
… of anxiety/worry, **boredom**/apathy and **flow** in their daily life, as … original quotations that **Csikszentmihalyi** extracted from the … of social isolation; physical **exercise** was practiced within the …

12.

Understanding **flow** experiences in professional athletes

AS Özdemir, TA Durhan - European Journal of Education Studies, 2020 - oapub.org

… **Flow** is what we call "autotelic experience" (**Csikszentmihalyi**… with **boredom**. One of the most
important contributions of … the dispositional **flow** according to gender and **exercise** behavior …

13.

24 hours on the Run—Does **boredom** matter for ultra-endurance Athletes' Crises?

C Weich, J Schüler, W Wolff - … of environmental research and public health, 2022 - mdpi.com

… This is also in line with research on **flow** (a state that is decidedly different from **boredom**), indicating that athletes can differ in their ability and frequency to **exercise** in a state of **flow** [52], …

14.

[HTML] Relationship between self-determination theory and **flow** in the domain of sports and academics among student-athletes

CKJ Wang, PAG Demerin - Asian Journal of Sport and **Exercise** Psychology, 2023 - Elsevier

… where dispositional **flow** was evaluated, this study yielded one **flow** score for the tendency to experience **flow** in sports and another for the tendency to experience **flow** in academics. …

15.

The role of recreation specialization and self-efficacy on life satisfaction: the mediating effect of **flow** experience

H Tian, W Zhou, Y Qiu, Z Zou - International Journal of Environmental …, 2022 - mdpi.com

… **Csikszentmihalyi** developed the concept of **flow** experience to … **bored** and frustrated while negotiating factors such as poor weather, injuries, or lack of a partner [32]. Experiencing **flow**, …

16.

Adaptive high-intensity exergaming: The more enjoyable alternative to conventional training approaches despite working harder

L Röglin, S Ketelhut, K Ketelhut, E Kircher… - Games for health …, 2021 - liebertpub.com

… **exercise** on a treadmill. Since **flow** is a strong contributing factor for enjoyment in gaming, 42,43 the study further compares the **flow** experience between the two **exercise** conditions. …
